# Removing co-occurring contaminants of arsenic and vanadium with full-scale arsenic adsorptive media systems

**DOI:** 10.2166/aqua.2021.148

**Published:** 2021

**Authors:** Thomas J. Sorg, Abraham S. C. Chen, Lili Wang, Darren A. Lytle

**Affiliations:** a Office of Research and Development, U.S. Environmental Protection Agency, Cincinnati, OH 45268, USA; b ALSA Tech, LLC, North Potomac, MD 20878, USA; c Office of Water, U.S. Environmental Protection Agency, Washington DC 20460, USA

**Keywords:** adsorptive media, arsenic, drinking water, multi-contaminants, vanadium

## Abstract

The U.S. Environmental Protection Agency conducted an Arsenic Demonstration Program (ADP) whereby 50 full, small-scale arsenic removal treatment systems were evaluated for removing arsenic to below the maximum contaminant level of 10 μg/L and their operating cost for a minimum of 1 year. The majority (28) of the systems installed were adsorptive media (AM) technology with the media replaced when exhausted. This paper reports on the results of two ADP projects and two laboratory rapid small-scale column tests (RSSCTs) using the iron-based media, Bayoxide E33 (E33) AM for the removal of arsenic (As) and the co-occurring contaminants (COCs) of vanadium and to a lesser degree fluoride (F) and nitrate (NO_3_). The ADP studies found that the AM effectively removed the COC of V, but with a lower removal capacity than of As. One ADP study found the AM to be ineffective for the removal of F and NO_3_. The RSSCT conducted on two other source waters also found vanadium to be removed by the same AM. The study results suggested the AM selectively sequence of As > V > F = N. The study also investigated the AM to achieve an As limit of 5 μg/L. The AM was found to reduce As to below 5 μg/L with around 30% shorter treatment run lengths.

## Graphical Abstract

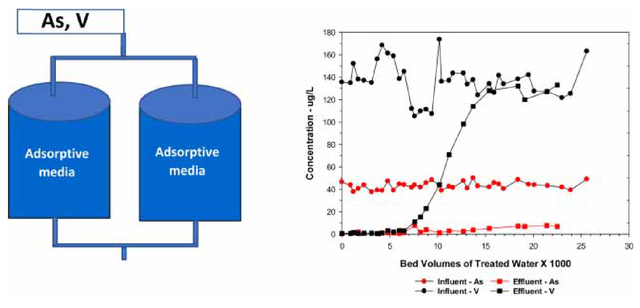

## INTRODUCTION

In January 2001, Environmental Protection Agency (EPA) lowered the maximum contaminant level (MCL) for arsenic in drinking water from 0.050 mg/L (50 μg/L) to 0.010 mg/L (10 μg/L). Because the lower arsenic MCL was expected to impact many small communities using groundwater and the lack of information on small system arsenic treatment technology, the EPA conducted 50 full-scale Arsenic Demonstration Program (ADP) treatment projects in 26 states (Sorg *et al.* 2015). The objective of this research program was to collect high-quality performance and cost data from the long-term (1–4 years) operation of a variety of treatment technologies to remove arsenic from groundwater. The technologies evaluated consisted of adsorptive media (AM), iron removal (IR), coagulation/filtration (C/F), ion exchange (IE), reverse osmosis (RO) and point-of entry (POE)/point-of-use (POU) RO. AM technology was installed at 28 sites (58%) because of its ease of operation and relatively low cost. Eight different AM products were utilized and all were listed by NSF International under Standard 61 for drinking water treatment. At the completion of each project, a comprehensive report was published by EPA that can be found at https://www.epa.gov/water-research/arsenic-treatment-technology-demonstrations.

In 2010, EPA proposed to address drinking water contaminants as groups rather than individually so that drinking water protection could be achieved more cost-effectively. The EPA initially targeted volatile organic compounds and nitrosamine disinfection by-products for regulatory groupings. EPA has also begun to identify other possible groupings including inorganic contaminants consisting of those already regulated, such as As, F, NO_3_, antimony (Sb) and uranium (U), and some of the inorganic contaminants on the EPA contaminant candidate lists 3 (EPA 2009) and 4 (EPA 2016), such as molybdenum (Mo) and vanadium (V). All of these inorganic contaminants occur in groundwater as anions and, therefore, have somewhat similar chemical characteristics to arsenic.

Because of the growing interest in technologies to remove multiple, co-occurring contaminants (COCs), a review was conducted of results of the EPA ADP projects. This review found that several of the ADP sites had source waters containing one or more co-contaminants that included F, Mo, NO_3_, Sb, U and V. Because of the lack of information on arsenic technologies to remove COCs, the authors published a paper on the removal of As and the COCs by IX, and POU/POU RO technologies from the performance data of four ADP projects (Chen *et al.* 2019). The review of the AM technologies projects found several projects to have their source water containing one or more COCs of F, NO_3_, Sb, U or V. Because of the extensive amount of data from these projects, this paper is limited to the results of two AM projects with V as the main COC and F, and NO_3_ as secondary COCs. Also included are the results of the rapid small-scale column test (RSSCT) on two other source waters of proposed ADP project sites having V as CCO. A companion paper is currently being prepared on the AM projects whose principle COC is uranium.

Of the nine different AM products installed by the 28 utilities, seven were iron-based or iron-modified products all developed for As removal (Sorg *et al.* 2015). Numerous controlled laboratory and pilot studies have been conducted on these AM products to determine the impact of the arsenic species (As(III)/As(V)) and concentration, source water pH, potential interference anions and system operating conditions on arsenic removal. The results of the iron-based media studies have clearly shown that the two most significant factors to affect performance are the As species and feedwater pH. Higher removal capacity is achieved on As(V) than As(III), and removal capacity increases with decreasing pH (Thirunavukkarasu *et al.* 2003; Amy *et al.* 2005; Saha *et al.* 2005; Xie *et al.* 2007; Guan *et al.* 2008).

Laboratory and pilot studies on potential interference anions to As removal found phosphate (PO_4_), silicate (SiO_2_) and vanadate (VO_4_) to have the most significant impact (Amy *et al.* 2005; Westerhoff *et al.* 2005; Xie *et al.* 2007; Möller & Sylvester 2008; Sperlich *et al.* 2008; Zeng *et al.* 2008; Nguyen *et al.* 2011). Sulfate (SO_4_) and chloride (Cl), commonly found anions in groundwaters, were not highly removed and therefore did not have a detrimental effect on arsenic removal (Sylvester *et al.* 2007). In these same studies, F and NO_3_ were not shown to be highly removed, although one laboratory study (Kumar *et al.* 2009) did show some removal of fluoride by the iron-based media, GFH. EPA has an F MCL of 4 mg/L and a NO_3_ (as N) MCL of 10 mg/L.

The main objective of this paper is to report on the effectiveness of two full-scale ADP AM systems and the results of RSSCT conducted on the source waters of two other proposed ADP project sites for the removal of As and V as a CCO. A published RSSCT laboratory study on V removal as a single contaminant found V to be effectively removed by two iron-based media products (Naeem *et al.* 2007). Although V is currently unregulated by EPA, it is included in EPA contaminant lists 3 and 4 for regulatory consideration and some states have issued drinking water guidelines ranging from 7 to 50 (Hazardous Substances Data Bank 2006). The removal of arsenic and the simultaneous removal of COCs, such as V, by full-scale treatment systems are totally lacking in the literature. Full-scale treatment systems operate under varying conditions, including feedwater quality, flow rates, daily use rates and occasional operation and maintenance problems, all of which can impact system performance. System performance in terms of media bed life (length of treatment runs) is significant because these AM products are used on a throw-away basis. The longer the bed life to contaminant breakthrough, the lower the operational cost. Full-scale performance data can support and verify the laboratory and/or pilot plant study data that are generally used for system design. The full-scale system studies become particularly valuable for small drinking water systems by increasing the confidence that these systems will perform as designed when operated under field conditions and assist in estimating operational costs of the technology. A secondary objective of this paper is to provide information on the removal of arsenic to ≤5 μg/L. An As limit of 5 μg/L has been a long time MCL of the State of New Jersey MCL and was signed into law by the Governor of the State of New Hampshire in 2019 and then adopted in the drinking water regulations in 2020 (State of New Hampshire 2020).

## MATERIALS AND METHODS

### ADP project sites and technologies

The site information and major design features of each treatment system of the two ADP study sites are provided in Table 1. Both treatment systems utilized the iron-based E33 AM, which was provided by Severn Trent Industries. The E33 media, used in 13 of the 28 AM projects, is classified as iron-based media (iron hydroxide/iron oxide) designed for arsenic removal (Amy *et al.* 2005; Westerhoff *et al.* 2006; Guan *et al.* 2008; Nguyen *et al.* 2011). The E33 physical and chemical properties are provided in Table 2. The source water quality of each ADP system, including the As and V concentrations, is shown in Table 3.

The two treatment systems were similar in design with each consisting of two down flow parallel pressure vessels. The design flow rates of both systems were slightly higher than the available well(s) total flow rates shown in Table 1. The estimated (media vendor) removal capacity to As breakthrough at 10 μg/L was significant different at 17,240 bed volumes (BV) for site 1 and 51,000 BV for site 2 (Table 1). The site 1 system designs empty bed contact times (EBCT) of 5.7 min, which was slightly higher than site 2 of 4.5 (Table 1). The average operating times (h/day) to meet the daily usage range were about the same 5.9 h (site 1) and 4.38 h (site 2) (Table 1). Both the EBCT and operating times have an impact on arsenic removal (Badruzzaman *et al.* 2004; Lee *et al.* 2004; Zeng *et al.* 2008). As removal capacity increases with increasing EBCT and decreasing operating time (increases system resting time). Because the pH was above 8.0 at site 2, the pH was adjusted down to 7.0 (CO_2_ gas injection system) to increase As removal as recommended by the equipment vendor.

### RSSCT studies

During the ADP studies, EPA funded several special RSSCT studies to evaluate the method for estimating the performance of various AM to remove As from source waters. The results of several preliminary As RSSCT studies indicated the method had the potential to predict the performance of full-scale systems (Westerhoff *et al.* 2005). The test locations included six ADP project sites, one ADP potential site and one EPA pilot-scale test site in Ohio. For each location, RSSCTs were conducted using three to four parallel columns packed with different AM following the procedures described in the final EPA report (Westerhoff *et al.* 2008). A review of these studies found two sites with elevated levels of V (37 and 34 μg/L) (Table 3).

### Sample collection and handling

During the initial site visit to each ADP site, an EPA contractor (Battelle, Columbus, OH) collected two sets of water samples of their source water well(s). One set of samples was analyzed on-site for pH, temperature, dissolved oxygen (DO) and oxidation—reduction potential (ORP). The second set was filtered using 0.45-μm syringe filters and the filtrate separated for soluble As(III) and soluble As(V) using a speciation method described in Sorg *et al.* (2014). These samples were analyzed by the Battelle laboratory or its sub-contract laboratories for arsenic and other selective parameters. During the ADP studies, water treatment plant operators were responsible for sample collection and the on-site water quality analyses after being trained by a Battelle staff member.

### Analytical methods

The on-site measurements of pH, temperature, DO and ORP on the well waters were conducted using a WTW Multi 340i handheld meter. The laboratory analytical methods were all EPA-approved methods as described in an EPA-endorsed Quality Assurance Project Plan (QAPP) (Battelle 2003). Although the majority of the test parameters were conducted on all water samples, a few of them (Sb, Mo, U and V) were not always included at each site because the initial measurement(s) of the parameter was found to be low and continuous monitoring was not considered to be of value. The method used for the metal analyses, including Sb, As, U and V, was inductively coupled mass spectrometry (ICP-MS); USEPA Method 200.8. Laboratory quality assurance/quality control (QA/QC) of all methods followed prescribed guidelines. The DL for As was 0.01 μg/L and for V was 0.1 μg/L.

## RESULTS AND DISCUSSION

### Source water quality

The source water quality of the ADP sites (Table 3) was based upon well water samples collected during the initial project site visits and was generally representative of the sampling results obtained during the long-term performance evaluation studies. Both treatment systems were supplied by multiple wells; site 1 having five wells and site 2 having two wells. Site 1 had the widest range in the system’s feedwater As and V concentrations (41 samples) with an As range of 6.0–50.6 μg/L (36.0 μg/L average) and a V range of 17.5–167 μg/L (112 μg/L average). The site 2 feedwater concentrations (28 samples) of As ranged from 29.0 to 38.6 μg/L (34.9 average). The source water As of the two sites was predominately As(V) (site 1 93% and site 2 97%) with some water containing traces of As(III). Both systems provided pretreatment chlorination for disinfection that converted any existing As(III) into As(V) (Frank & Clifford 1986; Dodd *et al.* 2006). Because the pH of site 2 was above 8 (8.2), it was adjusted down to 7.0 to increase As removal capacity. No change was made to the value of pH 7.7 of site 1.

Site 1 had significantly higher source water levels of F (5.3 mg/L) and NO_3_ (5.4 as N) than the site 2 levels of F (0.6 mg/L) and NO_3_ (1.1 as N). The removal of the F or NO_3_ was not expected at either site. SiO_2_ concentrations were 45.9 and 26.4 mg/L for sites 1 and 2, respectively. Neither site had a detectable level (0.06 μg/L) of P.

### ADP systems operation

Water utility staff operated the treatment systems after being trained by the equipment vendors. In contrast to controlled laboratory or pilot plant studies, full-scale systems will often experience operational and mechanical variations and issues that can influence system performance. When operational problems occurred, the operators were instructed to contact Battelle project personnel, who, with input from the equipment vendor as needed, provided advice and/or assistance to correct the issues.

#### Site 1 system results

**Figure 1 |:**
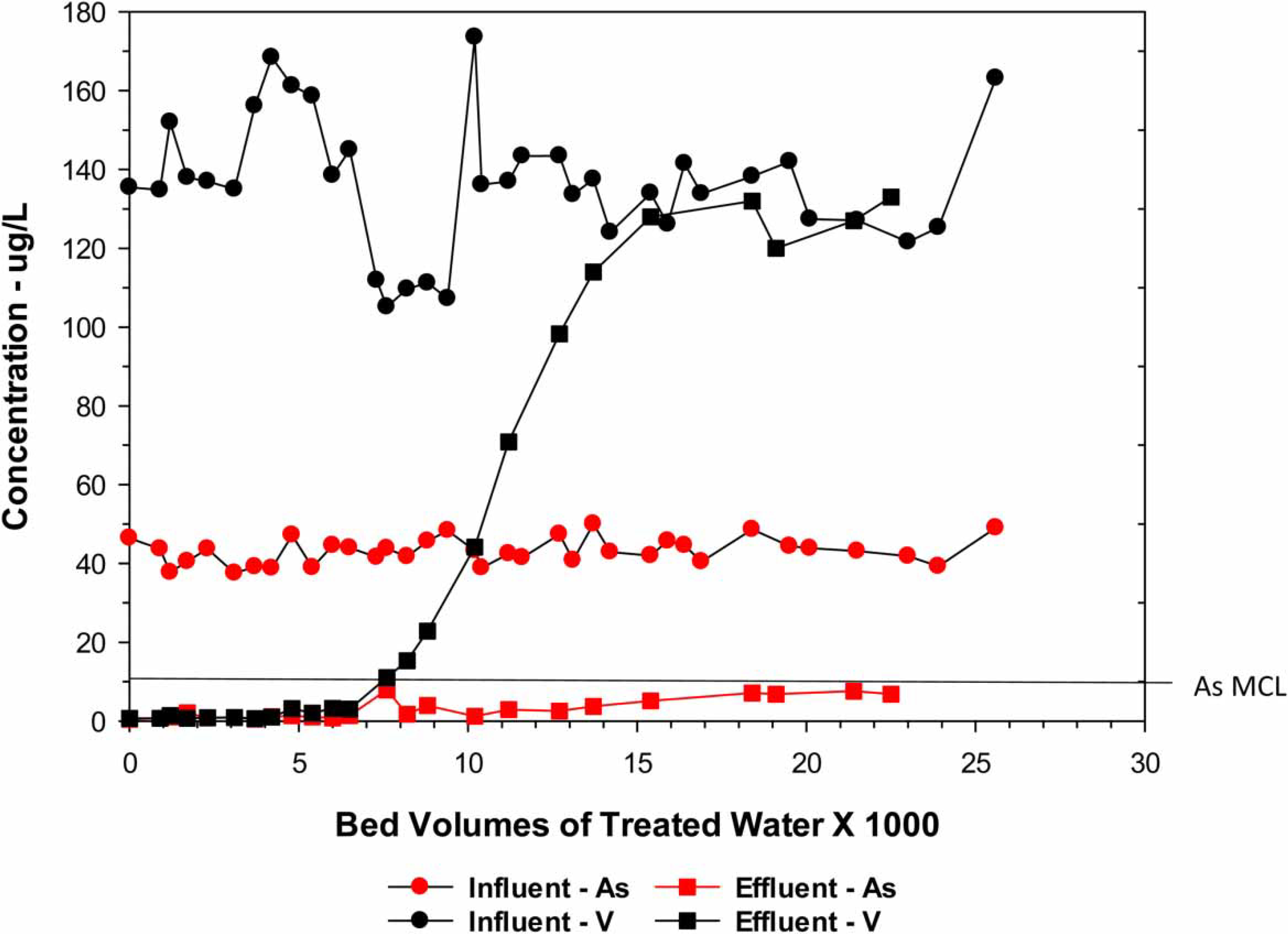
Performance of the site 1 AM system for the removal of arsenic and vanadium.

The performance evaluation study was conducted for a 21-month period with the system operating for 3,615 h for an average of 5.9 h/day or an utilization rate of 24%. Water samples were collected and analyzed before and after the treatment system (three) every other week for a total of 41 sets of samples. Because of flow differences experienced between the two treatment vessels, some differences in the As and V effluent levels occurred. The results of the As and V analyses (composite for the effluent from the two tanks of media) are presented in Figure 1. The As effluent water was not detected (<1 μg/L) up to around 4,000 BV of treated water when it began to increase reaching 5 μg/L around 15,000 BV of treated water. When the study ended at 22,500 BV, the As level was 7 μg/L which was above the vendor’s estimated As removal capacity at 10 μg/L.

The effluent V began to increase from around 1 μg/L at 7,000 BV and reach a total breakthrough of around 15,000 BV. At this same point, the As effluent was around 4 μg/L, indicating that As was more preferred than V. Depending on a V effluent goal, V would be the controlling contaminant over arsenic. Although some SiO_2_ (approximately 50%) was removed during the first 1,000 BV, silicate remained relatively constant across the treatment train, averaging 45.2 mg/L. The E33 media was found to be totally ineffective for the removal of F and NO_3_. Of these two test results, F was the most significant because the source water concentration average was 5.5 mg/L, which is above the MCL of 4 mg/L.

#### Site 2 system results

The performance evaluation study was conducted for a 25-month period with the system operating for 3,353 h for an average of 4.3 h/day or an utilization rate of 18%. Because the pH was above 8.2, the pH was adjusted down to 7.0 to increase As removal. During the startup period, some problems with the pH adjustment CO_2_ gas flow system occurred. The problems were corrected and the system functions properly throughout the remaining study period.

**Figure 2 |:**
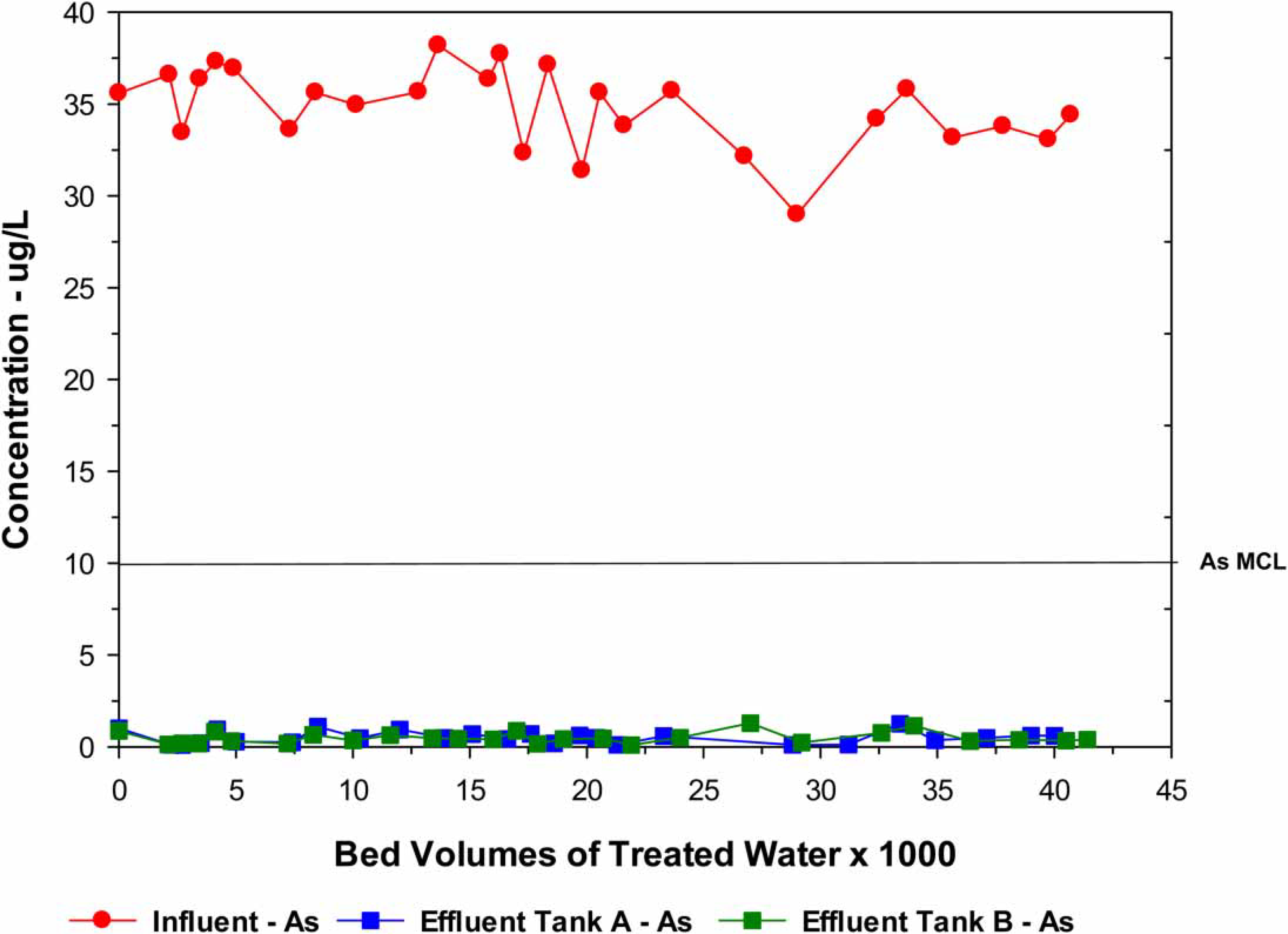
Performance of the site 2 AM system for the removal of arsenic.

**Figure 3 |:**
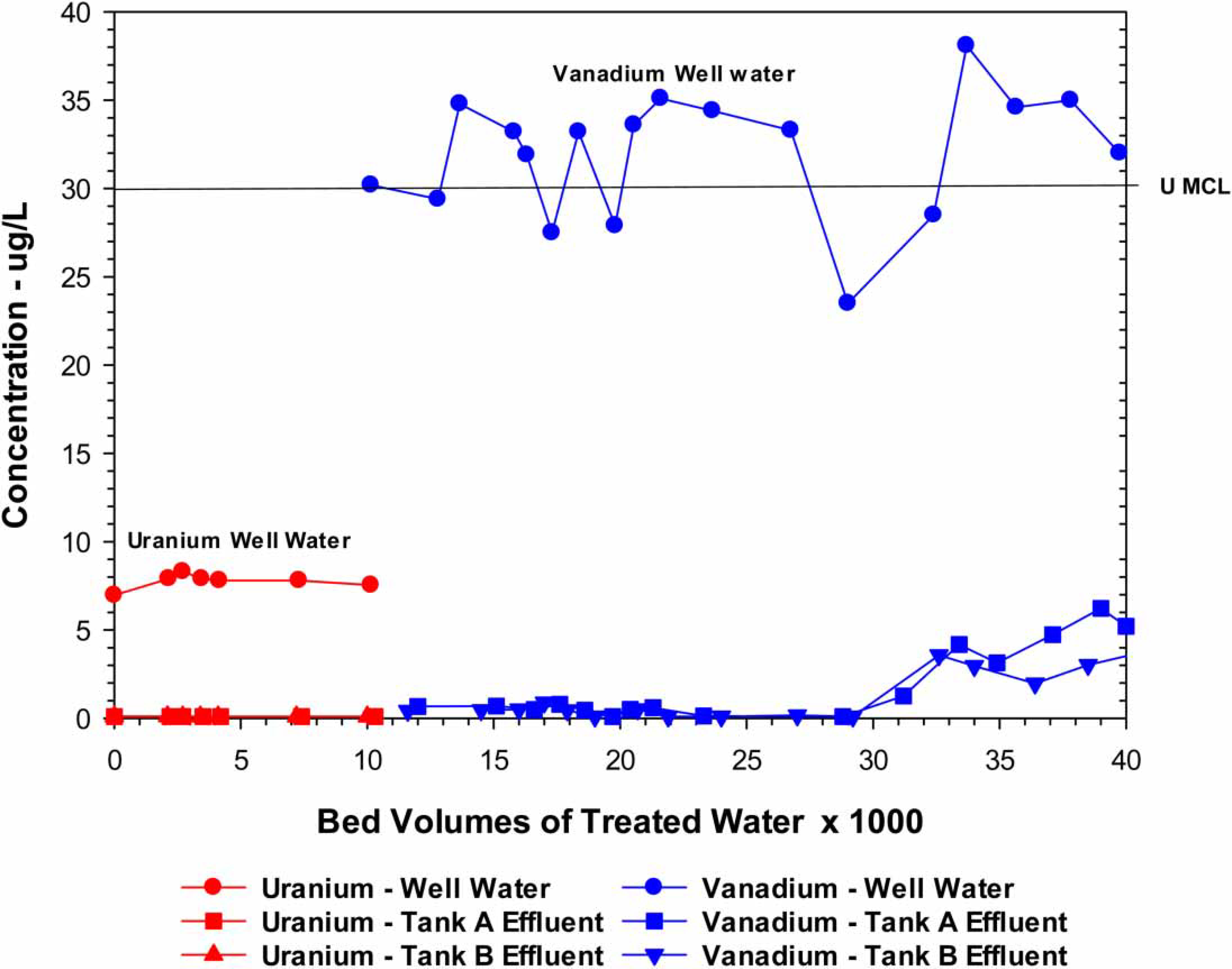
Performance of the site 2 AM system for the removal of uranium and vanadium.

Water samples were collected and analyzed before and after the treatment system (both vessels) once a month on average for a total of 28 sets of samples. The treatment system was extremely effective in removing the As to <1 μg/L during the entire evaluation period (41,000 BV) (Figure 2). In addition to As, the source water contained U (7.7 μg/L) and V (32.2 μg/L). During the first 9 months, seven sets of uranium sample were collected and analyzed. Because the source water levels were below the U MCL of 30 μg/L and the treated water samples were >0.1 μg/L, U analysis was discontinued in favor of V with a significantly higher source water level of around 31 μg/L. The V effluent level was <1 μg/L up to around 28,000 BV at which time they began to increase reaching 5.3 and 3.3 μg/L after Vessels A and B, respectively, at 41,000 BV (Figure 3) when the study ended. F, NO_3_ and SiO_2_ were essentially unchanged during the entire study. The As and V results clearly indicated that lowering the pH had a substantial positive effect on removals.

### RSSCT results

The RSSCT conducted on the site 3 source water (As 22.5 μg/L; pH 7.7) found the As effluent reached 5 μg/L at 18,000 BV and 10 μg/L around 25,000 BV. The test results for V (37 μg/L) showed effluent V to be >1 μg/L up to 15,000 BV when it increased to 10 μg/L at around 21,000 BV. The V results were less than the As results with the total V breakthrough (at 37 μg/L) occurring around 41,000 BV.

The RSSCT results on the site 4 source water (As 27.6 μg/L; pH 8.02) showed the As effluent reaching 5 μg/L around 26,000 BV and 10 μg/L around 40,000 BV. The test results for V (34.4 μg/L) showed effluent V to be >1 μg/L up to 15,000 BV when it increased to 10 μg/L at around 33,000 BV. Once again, the V results were slightly less than the As results.

## CONCLUSIONS

Controlled laboratory and pilot studies are usually designed to investigate the effect of one design parameter on system performance and the information is useful in system design and operation. These types of studies are less costly and less time-consuming than full-scale system studies. Full-scale studies, on the other hand, rarely have any fixed system variables, are very costly and take many months to years to complete. The usefulness or practical value of full-scale system studies, however, is that they provide the true-life performance of a system when water quality and system operation variables are not well controlled.

The results of the two ADP AM full-scale system studies supplement by two RSSCT results on source waters contain As and V, which have led to the following conclusions:

1. E33 AM treatment systems designed for arsenic removal are capable of simultaneously removing the CCO of vanadium, but ineffective for F and NO_3_ removal.
2. The breakthrough results of As, V, F and NO_3_ of the E33 AM studies indicate the media selectively (ion-preferred) sequence as follows:
  $$\text{As}(\text{V})>\text{V}>\text{F}={\text{NO}}_{3}$$
3. The E33 AM is capable of producing an As effluent of 5 μg/L. Based upon the RSSCT results, the length of treatment runs would be reduced to around 30% from what could be achieved if the media change out limit is 10 μg/L. Because AM technology is currently used on a throw-a-way basis when the media is exhausted, the cost of meeting a 5-μg/L limit could be cost-prohibitive for some small systems. To reduce the operational costs, pH adjustment should be considered.

## Figures and Tables

**Table 1 |:** ADP site and system design information

	Site 1	Site 2

Site information		
Location	Wellman, TX	Tohono O’odham Nation, AZ
Type of system	Municipal	Municipal
Population served	225	310
No. of well(s) used for ADP	9	2
Available flowrate for ADP (gal/min; L/min)	90; 341	60; 227
Average daily demand (gal/day; m^3^/day)	50,000; 189	36,000; 136
COCs	V, F, NO_3_	V, F, NO_3_
System design		
No. of vessels and size (in; cm)	2 × (48 D × 72 H); 2 × (122 D × 183 D)	2 × (36 D × 72 H); ×(91 D × 183 D)
Vessel configuration/construction	Parallel/steel	Parallel/FRP
Media	E33(P)	E33(P)
Volume (ft^3^/vessel; m^3^/vessel)	38; 1.08	19; 0.54
Design flowrate (gal/min; L/min)	100; 379	64; 242
Hydraulic loading rate (gal/min/ft^2^; L/min/m^2^)	4; 163	4.4; 179
EBCT (min)	5.7	4.5
Estimated working capacity (BV) to As at 10 μg/L	17,240	51,000
Vendor estimated media life (months)	10.1	21

**Table 2 |:** Physical and chemical properties of Bayoxide E33 AM^a^

Physical properties		Chemical properties	
Parameter (unit)	Value	Constituents	Weight (%)

Matrix	Iron oxide composite	FeOOH	90.1
Physical form	Dry granular media	CaO	0.27
Color	Amber	SiO_2_	0.06
Bulk density (lb/ft^3^; g/cm^3^)	35; 056	MgO	1.00
BET area (m^2^/g)	142	Na_2_O	0.12
Attrition (%)	0.3	SO_3_	0.13
Moisture content (%)	<15 (by weight)	Al_2_O_3_	0.05
Particle size distribution (U.S. standard mesh)	10 × 35	MnO	0.23
Crystal size (Å)	70	TiO_2_	0.11
Crystal phase	*α* – FeOOH	P_2_O_5_	0.02
		Cl	0.01

a *Source*: Bayer AG.

BET, Brunauer, Emmett and Teller.

**Table 3 |:** Source water quality parameters of study sites

		Site 1^a^	Site 2	Site 3	Site 4
	
Chemical contaminants	Units	ADP study sites^b^		RSSCT study sites	

As (total)	μg/L	45.4	32.5	21.5	27.6
F	mg/L	5.3	0.6	0.1	0.08
NO_3_-N	mg/L	5.4	1.1	NA	NA
U	μg/L	10.1	7.7	40	NA
V	μg/L	145	31.5	37	34.4
Common chemical parameters					
Cl^−^	mg/L	75	21	36	5.7
Fe	μg/L	<25	<25	38	NA
Mn	μg /L	2.0	0.8	0.07	NA
P	μg/L	<0.06	<0.06	54	NA
SiO_2_	mg/L	45.9	26.4	NA	28.4
SO_4_	mg/L	240	23	476	29.9
TDS	mg/L	823	231	NA	NA
TOC	mg/L	3.4	1.0	3.8	NA
pH	Unit	7.7	8.2	7.7	8.02
Alkalinity as CaCO_3_	mg/L	250	150	342	121
Hardness as CaCO_3_	mg/L	112	39	NA	NA

a Five wells combined chlorinated water.

b Test results of water samples from initial site visits.

NA, not analyzed.

## Data Availability

All relevant data are included in the paper or its Supplementary Information.

## HIGHLIGHTS

- The iron-based adsorptive media (AM), E33, designed for arsenic removal, are also effective for removing vanadium as a co-occurring contaminant, but with a lower removal capacity than for As.
- The iron-based AM, E33, are capable of reducing arsenic to less than 5 μg/L with around 30% shorter treatment run lengths than that achieved with a cut-off limit of 10 μg/L.
